# Processing of Aluminium-Silicon Alloy with Metal Carbide as Reinforcement through Powder-Based Additive Manufacturing: A Critical Study

**DOI:** 10.1155/2022/5610333

**Published:** 2022-01-10

**Authors:** R. Raj Mohan, R. Venkatraman, S. Raghuraman, P. Manoj Kumar, Moti Lal Rinawa, Ram Subbiah, B. Arulmurugan, S. Rajkumar

**Affiliations:** ^1^School of Mechanical Engineering, SASTRA Deemed to be University, 613401, Thanjavur, Tamil Nadu, India; ^2^Department of Mechanical Engineering, KPR Institute of Engineering and Technology, 641407, Coimbatore, Tamil Nadu, India; ^3^Department of Mechanical Engineering, Government Engineering College, 326023, Jhalawar, Rajasthan, India; ^4^Department of Mechanical Engineering, Gokaraju Rangaraju Institute of Engineering and Technology, 500090, Hyderabad, Telangana, India; ^5^Department of Mechanical Engineering, Faculty of Manufacturing, Institute of Technology, Hawassa University, Ethiopia

## Abstract

Powder-based additive manufacturing (PAM) is a potential fabrication approach in advancing state-of-the-art research to produce intricate components with high precision and accuracy in near-net form. In PAM, the raw materials are used in powder form, deposited on the surface layer by layer, and fused to produce the final product. PAM composite fabrication for biomedical implants, aircraft structure panels, and automotive brake rotary components is gaining popularity. In PAM composite fabrication, the aluminium cast alloy is widely preferred as a metal matrix for its unique properties, and different reinforcements are employed in the form of oxides, carbides, and nitrides. However, for enhancing the mechanical properties, the carbide form is predominantly considered. This comprehensive study focuses on contemporary research and reveals the effect of metal carbide's (MCs) addition to the aluminium matrix processed through various PAM processes, challenges involved, and potential scopes to advance the research.

## 1. Introduction

**Figure 1:**
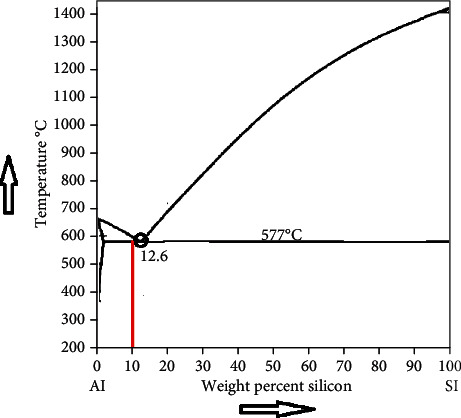
Equilibrium diagram of Al-Si alloy [12].

Powder-based additive manufacturing (PAM), also known as additive fabrication, processes the metal powders in an enclosed purged chamber and follows the layer deposition approach [1]. The development of lightweight aluminium-based composite and desirable properties is possible through the PAM process [2]. Generally, PAM follows two process routes, namely, Direct Energy Deposition (DED) with a laser energy source and powder bed fusion (PBF) with laser and the electron beam as an element of source [3]. For routes mentioned above, the raw materials (aluminium alloy) are in powder form with a spherical shape due to powder flowability [4, 5], and specified particle size ranges from 20 to 63 microns in PBF and 20 to 200 microns in DED. The PBF has the following advantages over DED: (i) excellent surface quality, (ii) high accuracy with precision, and (iii) a low dilution rate [6]. Laser source is chosen to melt/fuse the raw material in both routes due to its excellent optical characteristics such as coherence and high input energy transfer to the selective region [7]. The raw materials for PAM are manufactured by mechanical alloying and atomization process, namely, centrifugal, water, and gas atomization [8, 9]. The input parameters, such as layer or deposition height, energy density, scanning strategy, and hatch spacing, strongly influence the printed specimen's surface and mechanical properties [10, 11]. The most commonly used primary aluminium alloy with desirable properties in PAM is AlSi10Mg (hypoeutectic cast alloy), and its equilibrium diagram is shown in Figure 1 [12]. At optimum input parameters, the AlSi10Mg specimen prepared by the PAM process exhibits high relative density, tensile strength, stiffness, and impact toughness compared to the A360 die-cast alloy [13–16]. The homogenization of matrix and reinforcement particles must ensure the proper packing density [17]. Through High-Energy Ball Milling (HEBM), the mechanical (yield strength and ultimate strength) and surface properties (hardness) of the aluminium-based composite are enhanced by varying milling time (input parameter) compared to pure samples [18], and the possibility of balling effect occurs due to direct mixing instead of ball milling [19]. In general, the dispersed reinforcement strengthened the matrix, and it should not react with the matrix phase. The strength of the composite should be maintained without distortion at high temperatures [20]. So, ultra-high-temperature ceramic reinforcements or transition metal carbide are preferred [21]. Secondary operations like heat treatment, shot peening, and Hot Isostatic Pressing (HIP) are also employed to enhance the composite properties [22–24].

Investigations are focusing on optimizing the input parameters for fabricating the aluminium-based metal matrix composite (Al-MMC) with a limited choice of metal carbides (MCs) and subsequent secondary operations. This study is aimed at critically reviewing the effect of different MCs with varying weight percentages (reinforcement) on Al-Si alloy processed through PAM and describes the challenges with contemporary research scope. Therefore, various engineering applications are foreseen further study on various transition metal carbides (TMCs) as secondary elements.

## 2. Materials

### 2.1. Aluminium-Silicon Alloy (Al-Si) as Matrix

Aluminium-based alloys are employed in engineering applications due to their cost to performance ratio. They are mostly considered a matrix element in composite fabrication due to their enhanced mechanical properties, improved wear rate, fracture toughness, and better dimensional stability. Powder-based additive manufacturing is a favourable technology to process aluminium-based composite because it overcomes the issues related to traditional methods like nonuniform distribution, wettability issues, limited features, size, and geometrical tolerance [25]. Moreover, the aluminium-based composite properties are influenced mainly by the processing methods like PAM processing parameters, microstructure, composition with varying percentages of reinforcement, particle size, agglomeration tendency, and secondary operations [26–28]. Aluminium-based matrix composites are employed in various engineering applications such as rails, marine, automobile, and construction materials for durability, high mileage, fuel efficiency, and optimum strength, respectively [29]. Aluminium- (Al-) Silicon (Si) cast alloy is mainly preferred among aluminium-based alloys for its fluidity and castable properties. While increasing the Si content in Al, the mechanical properties tend to decrease [30] with the increase in tribological characteristics [31] like wear resistance and high coefficient of friction (CoF) as well as superior microhardness [32] and also, fine pseudoeutectic structure is observed which influences the strengthening of the specimen [33–35].

Further, the hypereutectic aluminium alloy possesses coarse silicon particles and causes adverse effects on the mechanical properties [36]. The hypoeutectic aluminium alloy (<12% of Si) is commonly used in PAM due to its high-temperature gradient, weldability and castability [37]. However, the mechanical properties of aluminium hypoeutectic alloy processed through PAM are comparatively lower than the cast route like friction stir processing [38]. A further reduction in aluminium alloy's silicon content leads to hot tearing during metal-based additive manufacturing (MAM) [39]. Besides, the eutectic composition of aluminium alloys processed through PAM shows that the partial melting leads to improper densification due to balling phenomenon [40, 41].  Table 1 shows the various types of aluminium alloy employed as a matrix element and preferable for composite fabrication.

**Figure 2:**
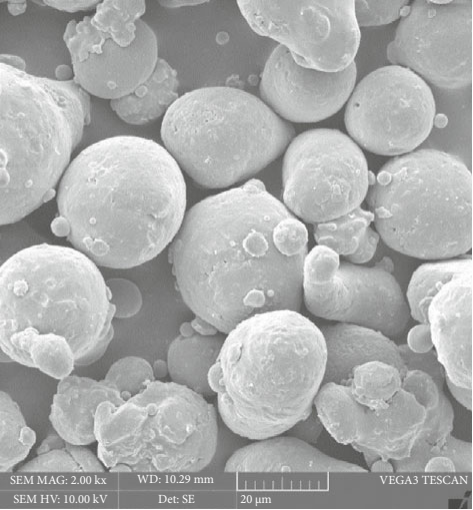
Surface morphology of AlSi10Mg powder.

The surface morphology of AlSi10Mg powder for PAM is shown in Figure 2. The spherical shape is generally favoured to enhance the flowability of the powders in AM machines.

### 2.2. Metal Carbides (MCs) as Reinforcement

Generally, metal carbides (MCs) are refractory materials (silicon carbide–IV A group and boron carbide–III A group), also known as high-temperature structural ceramics (HTSC), that can withstand high temperatures and extreme environmental conditions while retaining mechanical, chemical, and physical properties. MC offers exceptional thermal shock resistance, toughness, modulus of elasticity, corrosion resistance, and microhardness [48]. Besides, TMC is categorized into three groups: IV B, V B, and VI B. The carbides of titanium (Ti), zirconium (Zr), and hafnium (Hf) come under the group of IV B [49]. Similarly, V B exhibited the carbides of vanadium (V), niobium (Nb), and tantalum (Ta) [50, 51] and produced through magnesiothermic combustion [52]. Besides, VI B displays that chromium (Cr), molybdenum (Mo), and tungsten (W) in carbides form as chromium carbide (Cr_3_C_2_), molybdenum carbide (Mo_2_C), and tungsten carbide (WC), respectively [53]. These materials have an assortment of covalent bonds, metallic bonds, and ionic bonds with the precipitation of intermetallic carbide phases together [54]. Typically, silicon carbide (SiC) has a high Young modulus, stiffness, and strength [55].

Boron carbide (B_4_C) exhibits greater strength, impact resistance, and chemical stability. And it can endure higher temperature than SiC [56].  Table 2 illustrates the commonly used metal carbides as reinforcement. Due to the formation of a stable phase with the matrix, titanium carbide (TiC), zirconium carbide (ZrC), hafnium carbide (HfC), vanadium carbide (VC), niobium carbide (NbC), and tantalum carbide (TaC) are the most commonly used reinforcement elements for elevated environmental conditions [57]. From Table 2, comparing the different groups of metal carbides, it was observed that HfC has a high melting point, WC has a high density, and TiC has a high hardness. It is noted that each TMC has unique characteristics, and the anticipated composite properties can be achieved by adding TMC as particulate reinforcement.

Further, the mechanical as well as the physical characteristic of the composites can be enhanced. Moreover, chromium and molybdenum carbides are preferred for the highly corrosive atmosphere. For energy storage applications, vanadium carbide is used. Furthermore, the carbides used in the application of engineering tools are tungsten and niobium. Moreover, chromium and molybdenum carbides are preferred for the highly corrosive atmosphere. For energy storage applications, vanadium carbide is used. Furthermore, tungsten and niobium carbides are employed as cutting tools for machining.

**Figure 3:**
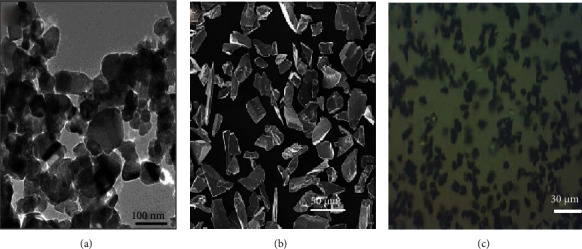
Morphology of (a) TiC [58], (b) SiC [64], and (c) B_4_C [66].

It was observed that titanium carbide (TiC), silicon carbide (SiC), and boron carbide (B_4_C) have played a significant role as a reinforcement in the MAM for processing Al-MMC than other MCs because of their wettability characteristics, size distribution, and good powder flowability. The morphology of the carbides as mentioned above is shown in Figures 3(a)–3(c).

**Figure 4:**
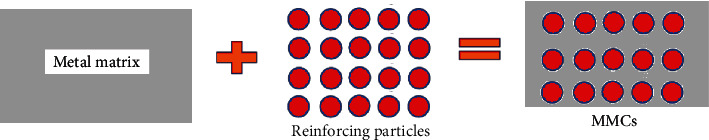
Illustration of MMC via ex situ route.

**Figure 5:**
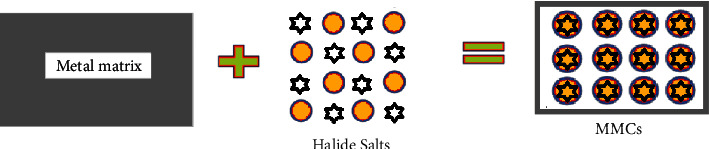
Illustration of MMC via in situ route.

Figures 4 and 5 show the illustration of MMC with uniform distribution of reinforcement (MC) in the ex situ route and in situ route, respectively [67]. The properties obtained through in situ fabrication were superior to those obtained from ex situ fabrication in terms of improved mechanical properties due to the formation of unique intermetallic phases. In both cases, increased particle size and applied energy transform the aggregation to the uniform dispersion of reinforcements.

## 3. Methods

### 3.1. Additive Manufacturing Techniques for Composite Fabrication

Powder-based additive manufacturing is initiated from the CAD (Computer-Aided Design) model in a digital format (Standard Tessellation Language (STL) extension), and the extension is accessible by the PAM equipment [38]. In PBF, the powder melted after spreading on the bed platform, whereas the powder melted while feeding through the multijet or coaxial nozzle in the DED [68–70]. The development of aluminium alloy-based composites through the powder bed fusion (PBF) process is attractive for high strength to weight ratio applications. In PBF, the selective laser melting (SLM) process uses a laser as a source in which the coherent beam of laser selectively traces and fuses the powder on the platform. The LASER variants employed in SLM are disk and fibre types. Optimizing input parameters of the SLM process [71] and decreasing the powder spread layer height lead to obtaining 100% full dense specimens with high ductility and mechanical strength [72].

Moreover, the scanning speed plays a significant role in densification and reducing cellular structure size [73, 74]. In laser-based powder bed fusion (L-PBF), Marangoni convection and recoil pressure are confounding factors during processing which cause denudation [75], spattering, and pores [76]. Because of their potential properties, the PBF is primarily used in the processing of composites.

**Figure 6:**
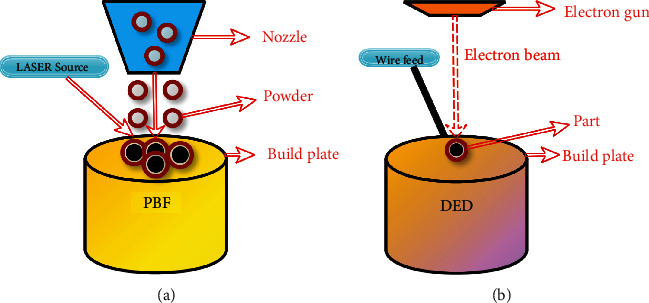
(a) PBF and (b) DED [88].

DED process is classified as Direct Light Fabrication (DLF), Laser Engineered Net Shape (LENS), and Laser Metal Deposition (LMD) based on the OEM (Original Equipment Manufacturer) specifications but the same working principle in both types. So far, Al-Si-Mg alloy, AA 6061, AA 2219, and AA 4047 are only aluminium-based materials applied in the DED process, and they are not employed for commercial purposes [77–84]. In the DED process, the fabrication of Al-MMC is possible rapidly [85] via a high deposition rate [86], and complex structures can be built, which exhibit excellent tribological properties. However, it has drawbacks like poor bonding between matrix and reinforcement, losing the MMC mechanical properties after adding ceramic reinforcement, high surface roughness, less deposition efficiency, less resolution in geometry, and formation of cracks due to temperature gradient [67]. The scanning speed and the curling effect, which forms the nonuniform structure, significantly influence the density and microhardness of the final part [87].  Figure 6 illustrates the process of PBF and DED. In PBF/DED, the fraction of liquid in the molten matrix pool affects the final microstructure and densification by altering the thermocapillary and thermokinetic nature.

## 4. Inferences and Discussion

The optimum conditions, surface characteristics, and reinforcement effect were discussed based on the previous sections of materials and methods. The optimum conditions of process parameters such as scanning speed, laser energy, layer thickness, bed temperature, and hatch spacing are mentioned in Table 3. For obtaining a fine microstructure, input parameter optimization is vital due to the ultraheating and cooling rate in the process. Furthermore, these parameters have an impact on the quality of the printed parts. It was found that the low-power laser power affects the melting of the matrix element, whereas the high laser power vaporizes the matrix element owing to variation in energy density. As a result, optimum laser power is required to melt the powders without the balling phenomena. In addition, increasing the scanning speed and hatch spacing reduces the energy density needed to fuse the powders. Furthermore, the powder deposition rate was decreased while the layer thickness was decreased. The optimum layer thickness controls the geometrical size of the depositing track on the build plate.

According to Table 4, the relative density of Al-MMC with varying percentages of reinforcement is greater than 95%, indicating that proper densification was achieved through the additive manufacturing process. The formation of new intermetallic phases increases the printed part hardness in SLM and DED. In comparison to Al-Si/TiC and Al-Si/B_4_C, the hardness of Al-Si/SiC was 217.4 HV at 15% of SiC in AlSi10Mg. Due to improper bonding between reinforcement and matrix element, the effect of B_4_C in hardness AlSi10Mg was relatively small.

From Table 5, the reinforcement TiC, SiC, and B_4_C with a varying percentage on the matrix of different aluminium alloy matrices display the effect on tensile strength, percentage elongation, coefficient of friction, and composite wear resistance. The wear resistance of the matrix was improved due to the hardness of TiC, SiC, and B_4_C. At different load conditions, the frictional force on the matrix was also lowered. The tensile property of the composite decreased as the percentage of reinforcement increased due to the formation of intermetallic phases.

However, the combination of Al-Si (matrix)+WC/VC/NbC/ZrC/Cr_3_C_2_/Mo_2_C/HfC/TaC (reinforcement) has not explored extensively through metal-based additive manufacturing. Physical, surface property, and wear studies were typically conducted on the specific combination of AlSi10Mg with TiC, SiC, and B_4_C. Furthermore, the investigation on corrosion and mechanical properties such as tensile strength, yield strength, and ultimate strength was not addressed extensively using the abovementioned combinations.  Table 6 consolidated the category of raw material, processing route with scanning mode, various MC, effect on characteristics of final specimen, and its applications. It was observed that the scanning strategy plays a concealed role in achieving good layer bonding during the printing process. The linear raster scan has good wettability and reinforcement distribution in the DED process due to a higher deposition rate [106], whereas the island and rotation of the 67° scanning strategy in SLM offer satisfactory performance in terms of wettability and adhesion. Compared to other alloys, the balling effect could be controlled while processing the eutectic aluminium-silicon alloy [77]. The energy absorption was increased when adding TiC and SiC with aluminium alloy except for B_4_C due to the formation of aluminium diboride (AlB_2_).

**Figure 7:**
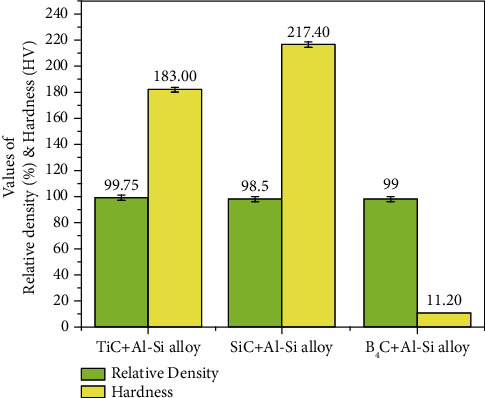
Maximum values of relative density and hardness.

**Figure 8:**
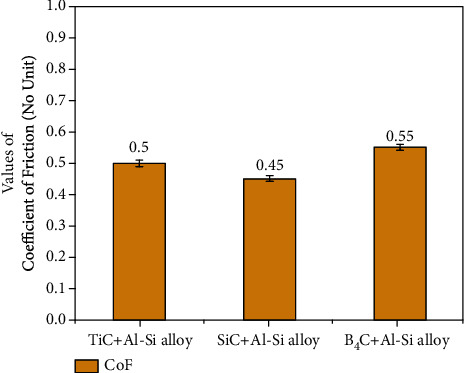
Minimum value of CoF.

Figure 7 shows the maximum relative density and hardness values obtained for different Al-Si alloy reinforcements based on the above literature. It was found that SiC has a high hardness but a lower relative density than TiC. In both responses, the result for B_4_C was lower.  Figure 8 depicts the influence of reinforcement on the coefficient of friction; it was observed that the frictional force induced by the reinforcement is significant against the counter body. As a result, the addition of reinforcement must be optimized to reduce frictional force.

Hence, TiC is an effective reinforcement candidate for Al-Si alloys due to its wettability, laser absorption, uniform reinforcement distribution, and increased mechanical properties. However, the coarsen TiC (reinforcement) particles are prone to splitting and spalling from the Al matrix during sliding. The descending order of reinforcement in terms of performance is as follows: TiC > SiC > B_4_C. Due to its brittleness, B_4_C seemed to have the slightest influence (needle-like structure).

### 4.1. Challenges and Potential Scope

The major challenge in processing aluminium-based alloys in SLM is reflectivity, low melting point, and interaction with oxygen in the environment [15, 38, 107–109]. Only 10% of the input energy is utilized to melt the powder, and the remaining is reflected. The preferred dimensional tolerance cannot be achieved due to the ultrarapid cooling cycle; thereby, shrinkage may occur [69, 110]. Moreover, the balling phenomenon occurs due to raw materials and processing conditions [111] due to irregularity in the scan track [112]. So, optimizing process parameters like scanning speed, hatch spacing, and layer height will solve these adverse effects. Also, the energy density influences the part quality. The microstructure of the SLMed part was different from the forged or cast part due to its complex processing mechanism [113]. While processing through the external addition method on SLM, the final parts may encounter pores, coarsening of grains, oxidation due to improper purging of inert gas in the building chamber [106, 114], and crack formation due to unmelted powder. Also, oxide formation cannot be eliminated while processing aluminium-based composites through SLM [115]. The final part distortion may happen in SLM and DED due to residual and thermal stress [116], and it can be sorted by process optimization [67]. In the AM process, the reinforcement addition with varying percentages can affect the solidification behaviour of the matrix composite [117]. The pool size and melt shape should be controlled to obtain good quality of the final printed specimen with specified microstructure and good surface finish [118–120]. The final printed sample can match the service requirements based on the selection of hatch spacing, preheating of the base plate, and contour [121]. Higher preheating temperature leads to sintering the powder particles with the base plate [122]. So, optimum preheating provides better control on balling effect.

**Figure 9:**
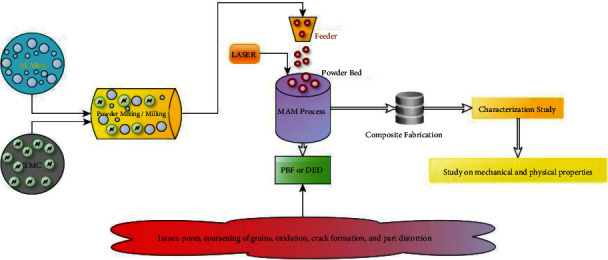
Workflow of the composite fabrication process and related issues.

Nevertheless, the solution to this effect has not been addressed. The postprocessing like heat-treatment like annealing [123] and T6 (aging followed by solution strengthening) [124], shot peening [125, 126], and sand-blasting [127] on aluminium-based composite may significantly influence the physical as well as mechanical properties of the specimen. Typically, the particles were coarsened, while heat treatment led to a lower hardness value [128]. Also, it consumes more time and is expensive [129]. Besides, the rejuvenating of nonconsumed powders again may raise issues like increasing oxygen content [130], change in morphology, and distribution of particle size [131]. Thus, proper characterization is required before using the nonconsumed powders [132, 133]. So, the appropriate process optimization and characterization reflect the characteristics of printed parts. Furthermore, the addition of reinforcement may decrease the reflectivity of the Al-Alloy and ease the processability [134] and also, submicron particles were more effective than micron size in the aspect of laser absorptivity [135]. But, agglomeration of particles may occur, which affects the mechanical properties of the composite [136]. Finally, the Al-TMC composites through MAM may create innovative changes in vital applications that require intricate, complex structures along with desirable properties. Also, the geometrical and microstructural features need to be investigated based on specimen orientation [137, 138]. Secondary reinforcements in Al-alloy may influence the grain refinement with high strength. The mechanical strengthening of the composites is possibly defined by the load-bearing transfer [139], the hall-patch effect [140], and the Orowan strengthening mechanism [141, 142]. Moreover, the impact of remaining TMCs as reinforcement in the aluminium matrix has not been much addressed, and a specific combination only exists (AlSiXX alloy+X% TiC) apart from SiC and B_4_C. The study on compressive strength, impact toughness, corrosion resistance, the outcome of built direction, and consequences of the subsequent heat treatment process for Al with TMC composites has received little consideration.  Figure 9 illustrates the workflow of composite fabrication (AlSi10Mg+TiC) and related issues involved.

## 5. Conclusions

This review has discussed many observations found in the recent research studies, such as aluminium alloys in additive manufacturing, metal carbides, various metal-based additive manufacturing processing techniques, the formation of intermetallic phases, and final properties of the aluminium-based composite by in situ and ex situ fabrication. From the review, the critical points were observed as follows:

1. The synthesis method of raw material, powder morphology, and reinforcement effect substantially influences composite fabrication's microstructure, surface integrity, and final properties
2. Overcoming the residual stress and ball effect phenomenon was challenging in composite additive manufacturing due to the ultracooling rate and inadequate linear energy density
3. Compared to conventionally processed composites, the additive manufactured composites positively influence the environment because of the wastage reduction and low energy consumption
4. Through metal-based additive manufacturing, processing all elements of TMCs is possible through either consolidation or coating approach for different applications
5. The novel Al-MMC should be developed to acquire a high-performance composite. Therefore, further exploration is essential to evaluate the process parameters, postprocessing, and different properties in the future

## Figures and Tables

**Table 1:** Various types of Al-Si alloys (matrix element).

Al alloy material (matrix)	Powder production method	Type of alloy	Ref.
AlSi10Mg	Gas atomization	Hypoeutectic	[37, 38] [41, 42]
AlSi20	Gas atomization	Hypereutectic	[30] [33, 34] [40]
AlSi30	Gas atomization	Hypereutectic	[35]
AlSi15	Gas atomization	Hyper eutectic	[31]
AlSi16	Mechanical alloying	Hypereutectic	[25]
AlSi50	Mechanical alloying	Hypereutectic	[43]
Al-303, Al-308, and A360	Mechanical alloying	Hypoeutectic	[39]
Al-356 and Al-357	Mechanical alloying	Hypoeutectic	[44–46]
A390 (18% weight of Si)	Mechanical alloying	Hypereutectic	[36] [47]

**Table 2:** Commonly used MC as a reinforcement.

MC	Density (g/cc)	Melting point (°C)	Hardness (HV)	Morphology	Property exhibits
Group IV B					
TiC	4.930	3066.9	3568.879	Irregular/polygonal [58]	Superior hardness
ZrC	6.730	3419	2640.971	Agglomerated [59]	Thermal stability
HfC	12.20	3920	2661.364	Dendrite [60]	High resistance to oxidation

Group V B					
VC	5.770	2649.5	2763.332	Irregular [60]	Grain growth inhibitor
NbC	7.820	3611	1998.572	Fragmented particles [61]	High wear resistance
TaC	14.30	3880	1702.865	Rippled surface [60]	High hardness

Group VI B					
Cr_2_C_3_	6.680	1809	1835.424	Spherical [62]	Low rate of oxidation
Mo_2_C	9.150	2519	2498.216	Irregular crystallites [62]	Exceptional thermal conductivity
WC	15.63	2775	2243.296	Irregular [63]	Increase the performance of wear and abrasion-resistant

Group IV A					
SiC	3.21	2731	2600	Irregular [64]	Good strength and high wear resistance

Group III A					
B_4_C	2.52	2763	3299	Fine particles and some nanoneedle structure [50, 65]	Wear resistance and high hardness, but brittle in nature

**Table 3:** Optimized conditions for aluminium-based (matrix) and MC (reinforcement) [31, 58, 66, 89–102].

ES	MAM-P	M-R	LP (W)	SS (mm/s)	LT (*μ*m)	HS (*μ*m)	BT (°C)
Laser	SLM	AlSi10Mg-3% TiC	80-140	200	50	50	100
Laser	SLM	AlSi10Mg-5% TiC	100	100-400	50	50	—
Laser	SLM	AlSi15-5% TiC	360	650	20	100	—
Laser	DED	AlSi10Mg-5% TiC	3000	10	—	2000	—
Laser	DED	AlSi10Mg-30 Vol% TiC	1800	7-17	—	—	100-200
Laser	SLM	Al9.8Si0.6MgTi- TiC	400	—	—	90	—
Laser	SLM	AlSi10Mg-5% TiC	320	1100	30	130	—
Laser	SLM	AlSi10Mg-15% SiC	500	600-2100	40	60-180	—
Laser	SLM	AlSi10Mg-5 Vol% SiC	195	640-880	30	500	—
Laser	SLM	AlSi10Mg-10 Vol% SiC	195	640-880	30	500	—
Laser	SLM	Al-15% SiC	500	—	50	100	—
Laser	Laser sintering	Al–7Si–0.3Mg—5 to 12 Vol % SiC	8.6	—	100	30	80
Laser	SLM	AlSi10Mg-20% SiC	200	100	30	50	—
Laser	SLM	Al7Si-10 Vol% SiC	200	500-1750	50	100	—
Laser	SLM	AlSi10Mg-20% B_4_C	100-200	100	50	130 to 150	—

ES: energy source; MAM-P: metal-based additive manufacturing; M-R: matrix-reinforcement; LP: laser power; SS: scanning speed; LT: layer thickness; HS: hatch spacing; BT: bed temperature.

**Table 4:** Relative density and microhardness of Al-MMC with MC [31, 66, 95, 97, 103–105].

MC	% weight	Matrix element	Relative density (%) (g/cc)	Hardness (HV)	Intermetallic phases
TiC	3	AlSi10	>96	183	Mg_2_Si and Al_9_Si
	5	AlSi10	>98.5	160-180	Mg_2_Si and Al_9_Si
	5	AlSi15	96.25	145-173	TiC
	5	AlSi10	95.8	139.1	Mg_2_Si and Al_9_Si
	10	AlSi15	98.5	177	TiC
	1 : 1	Al9.8Si0.6MgTi	99.7	—	Si_4_Ti_5_ and *α*-Al dendritic
	5	AlSi10Mg	99.75	131	D_022_-Al_3_Ti

SiC	15	AlSi10Mg	97.7	217.4	Mg_2_Si and Al_4_SiC_4_
	5	AlSi10Mg	98.5	—	*α*-Al dendritic network
	10	AlSi10Mg	98	—	Al_3.21_Si_0.47_
	15	Pure Al	92	140	Al_4_C_3_
	5-15	Al7Si0.3Mg	90	—	Al_4_SiC_4_
	20	AlSi10Mg	97.5	218.5	Al_4_SiC_4_
	10	Al-12Si	97.4	—	Al_4_C_3_

B_4_C	20	AlSi10Mg	97-99	11.2	Al_4_C_3_ and AlB_2_

**Table 5:** Effect of MC on mechanical properties of Al-Si alloy.

MCs with % of weight/volume	Matrix	Wear resistance	Coe. of friction	Tensile property	% elongation	Process
TiC—30% Vol.	AlSi12	++	∗	∗	∗	DED
TiC—3% Wt.	AlSi10Mg	+	-	+	+	SLM
TiC—5% Wt.	AlSi10Mg	+	-	++	+	SLM
TiC—5% Wt.	AlSi10Mg	+	-	+	+	DED
TiC—5% Wt.	AlSi15	+	-	+	+	SLM
TiC—10% Wt.	AlSi15	+	-	--	-	SLM
TiC—1 : 1	Al9.8Si0.6MgTi	∗	∗	+	+	SLM
TiC—5% Wt.	AlSi10Mg	∗	∗	++	++	SLM
SiC—15% Wt.	AlSi10Mg	+	∗	-	-	SLM
SiC—5% Vol.	AlSi10Mg	∗	∗	∗	∗	SLM
SiC—10% Vol.	AlSi10Mg	∗	∗	∗	∗	SLM
SiC—15% Wt.	Al	+	-	∗	∗	SLM
SiC—5 to 15% Vol	Al–7Si–0.3Mg	∗	∗	∗	∗	Laser sintering
SiC—20% Wt.	AlSi10Mg	++	--	∗	∗	SLM
SiC—12% Vol.	Al-12Si	∗	∗	∗	∗	SLM
B_4_C—20% Wt.	AlSi10Mg	+	-	∗	∗	SLM

+, increase; ++, drastic increase; -, decrease; --, drastic decrease; ∗, no observation.

**Table 6:** Consolidation of MC effect on the aluminium-silicon alloys with applications through additive manufacturing.

Category	MC	Route	Process	Scanning mode	Characteristics			Purpose	Ref.
					Wettability	Reinforcement distribution	Energy absorption		
Eutectic aluminium alloy	TiC	Ex situ	SLM	Islands	Good	Good	Increased	Aerospace	[101]
Eutectic aluminium alloy	TiC	In situ	SLM	Rotation of 67°	Good	Good	Increased	Biomedical	[102]
Eutectic aluminium alloy	TiC	Ex situ	SLM	Linear raster scan	Good	Good	Increased	Microelectronics	[58]
Hypereutectic aluminium alloy	TiC	In situ	SLM	Long bidirectional	Excellent	Homogenous	Increased	Automotive	[91]
Eutectic aluminium alloy	TiC	In situ	DED	Linear raster scan	Good	Good	Increased	Aerospace, automotive, and biomedical	[106]
Eutectic aluminium alloy	SiC	Ex situ	SLM	Alternating *x*/*y* raster strategy	Good	Uniform	Increased	Automotive, military, aerospace, and electronic encapsulation fields	[95]
Eutectic aluminium alloy	SiC	Ex situ	SLM	Single-line tracks	Good	Uniform	Increased	Specialized products	[96]
Pure aluminium	SiC	Ex situ	SLM	Style of strip hatch (17°)	Good	Uniform	Increased	Automotive and aerospace sectors	[97]
Hypoeutectic aluminium alloy	SiC	Ex situ	Laser sintering	Linear	Fair	Agglomeration	Increased	Aerospace applications	[98]
Eutectic aluminium alloy	SiC	In situ	SLM	Alternate *XY* directions	Better	Homogenization	Increased	Tribological application	[99]
Hypereutectic aluminium alloy	SiC	Ex situ	SLM	Series	Good	Uniform	Increased	Marine, automotive, and aerospace	[100]
Eutectic aluminium alloy	B_4_C	Ex situ	SLM	Bidirectional	Fair	Uniform	Reasonable	Radial collimators, lightweight armor	[66]

## Data Availability

The data used to support the findings of this study are included within the article.
